# Contralateral Axillary Metastasis With Synchronous Ipsilateral Axillary Metastasis From Occult Breast Cancer: A Case Report With a Review of the Literature

**DOI:** 10.7759/cureus.100096

**Published:** 2025-12-25

**Authors:** William Swee Keong Khoo, Rasika Hendahewa

**Affiliations:** 1 Department of General Surgery, Townsville Hospital, Townsville, AUS; 2 Department of General Surgery, Caboolture Hospital, Brisbane, AUS

**Keywords:** breast cancer, contralateral axillary metastasis, general surgery, metastatic breast cancer, occult breast cancer

## Abstract

Occult breast cancer (OBC) is a rare presentation of breast cancer, characterised by evidence of metastasis to axillary lymph nodes without a clear radiological lesion in the breast. Contralateral axillary metastasis (CAM) in breast cancer is another rare presentation, with various differentials. This case report presents a 67-year-old female with left axillary metastasis from an OBC with synchronous CAM. This case highlights the complexities of both rare presentations in a single patient and discusses the pertinent therapeutic recommendations from current literature and guidelines.

## Introduction

Occult breast cancer (OBC) is a rare presentation of breast cancer, characterised by breast carcinoma that has metastasised to the axillary lymph nodes, with the absence of radiological disease in the breast [1-3]. Contralateral axillary metastasis (CAM) in breast cancer is another rare occurrence, with differentials including contralateral metastasis from the primary breast cancer, metastasis from a separate ipsilateral primary breast cancer, or metastasis from another primary cancer of extramammary origin [4].

This case presents a 67-year-old female with left axillary metastasis from an OBC with synchronous CAM. Her treatment included neoadjuvant chemotherapy (NACT), bilateral mastectomy with bilateral axillary lymph node dissection (ALND), and adjuvant radiotherapy. This case highlights the complexities of both rare presentations in a single patient and discusses the pertinent therapeutic recommendations from current literature and guidelines.

## Case presentation

A 67-year-old woman was referred to the General Surgery Breast Clinic with a five-week history of left axillary swelling. The patient had a history of hypertension, a benign breast lumpectomy five years ago, and a hysterectomy at 34 years of age. She has no known family history of breast or ovarian cancer, is an ex-smoker of 20 pack-years, and drinks alcohol regularly. Her gynaecological history includes menarche at the age of 15 years, no previous contraception use, and she has never breastfed. A bedside examination revealed palpable left axillary lymphadenopathy, with no palpable breast lumps bilaterally.

**Figure 1:**
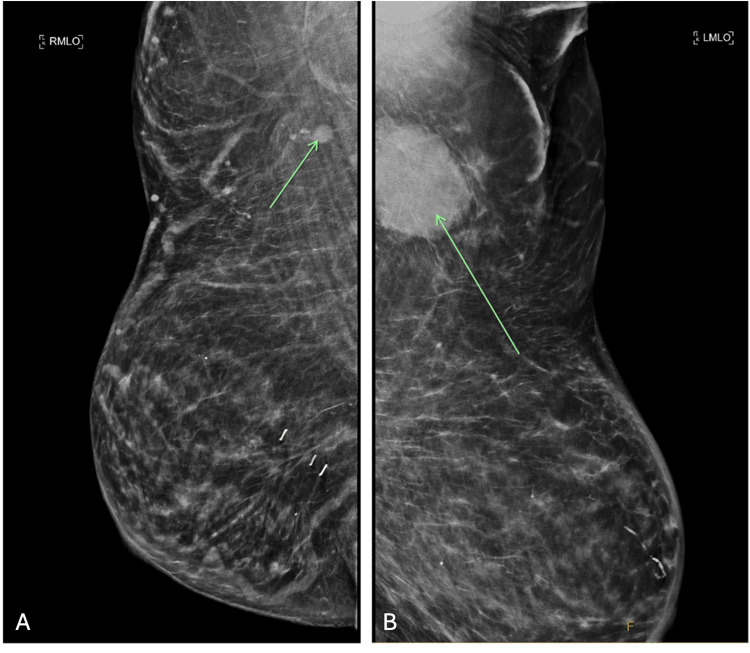
(A) Right breast MLO view and (B) Left breast MLO view Mammogram demonstrating bilateral axillary lymphadenopathy suspicious for metastasis as indicated by arrows. MLO, median lateral oblique

An initial workup with breast ultrasound and mammogram revealed suspicious pathological lymphadenopathy in the bilateral axillae (Figure 1). However, no obvious changes were identified on the mammogram, aside from mild dermal thickening of the left breast, which was benign on punch biopsy of the left breast skin. A fine-needle aspirate (FNA) of the largest left axillary lymph node revealed pleomorphic lobular carcinoma with changes consistent with metastatic breast carcinoma, which was estrogen receptor (ER) negative, progesterone receptor (PR) negative, and human epidermal growth factor receptor 2 (HER2) positive.

**Figure 2:**
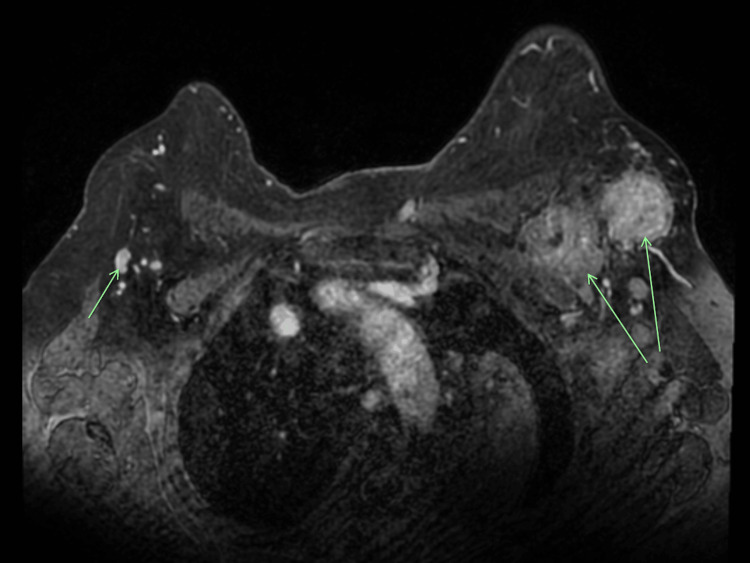
Axial view of MRI Axial view of MRI, demonstrating bilateral axillary lymphadenopathy (indicated with arrows), without evidence of pathological lesions in either breast. MRI, magnetic resonance imaging

**Figure 3:**
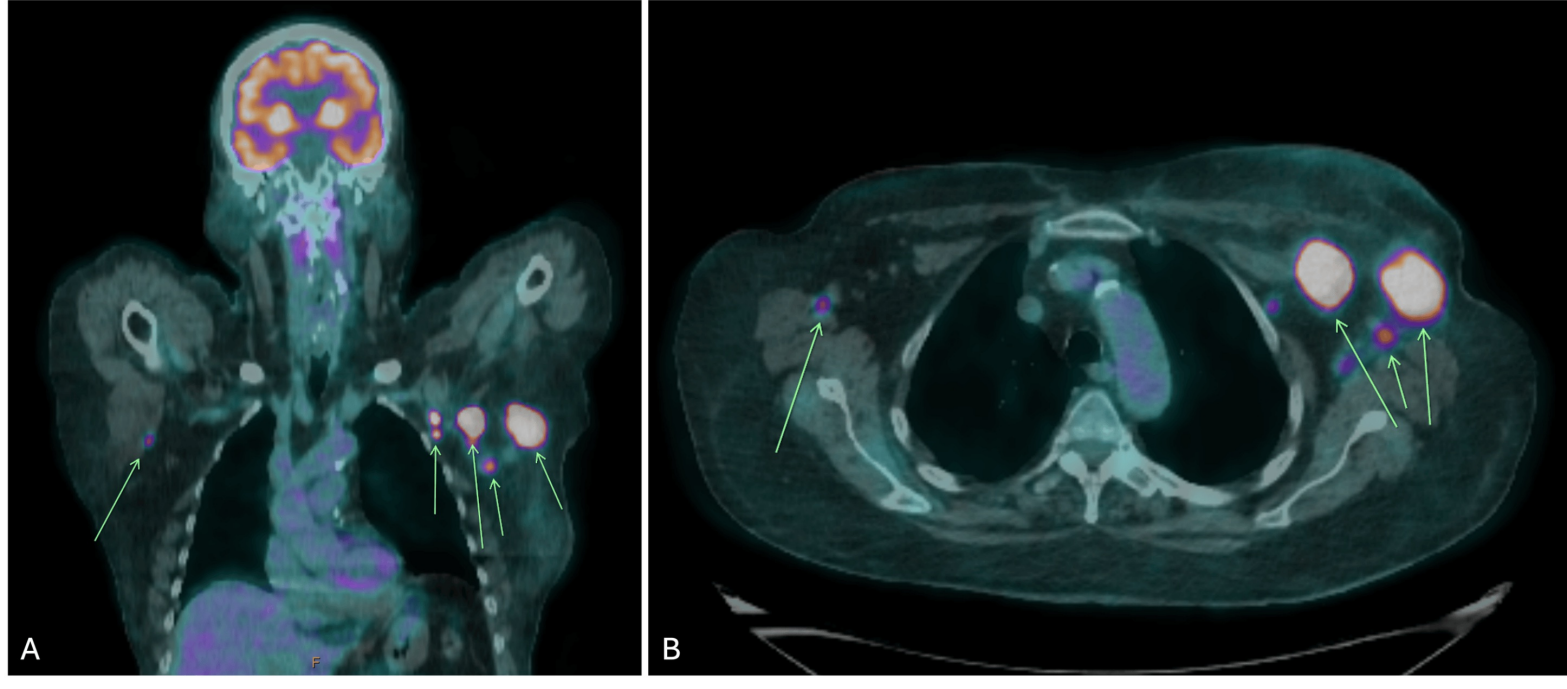
(A) Coronal view and (B) Axial view PET scan demonstrating intense abnormal FDG uptake, consistent with metastatic left axillary lymphadenopathy, with a focus of FDG avidity in the right axilla, suspicious for contralateral axillary lymph node metastasis (as indicated by arrows). PET, positron emission tomography; FDG, fluorodeoxyglucose

Subsequent investigations with a breast magnetic resonance imaging (MRI) revealed evidence of bilateral lymphadenopathy, with no clear breast lesions identified (Figure 2). Whole-body positron emission tomography-computed tomography (PET-CT) revealed intense abnormal fluorodeoxyglucose (FDG) uptake, consistent with metastatic lymphadenopathy in the left axilla and clavipectoral region, with a small FDG-avid focus in the right axilla, suspicious for contralateral axillary lymph node metastasis (Figure 3). Additionally, FNA biopsy of the right axillary lymph node demonstrated metastatic pleomorphic lobular carcinoma, which was ER and PR negative, but HER2 positive.

After discussion of the patient’s case in the Breast Surgery Multidisciplinary Team (MDT) meeting, the decision was made for NACT with carboplatin/doxetaxel and Herceptin. A follow-up staging CT and bilateral breast and axilla ultrasound scan (USS), three months post-initial workup, demonstrated a favourable treatment response of the bilateral axillary lymphadenopathy, without further evidence of progression of metastatic disease. A core biopsy of a newly identified left breast lump at 3 o’clock, 3 cm from the nipple, was conducted but demonstrated benign breast parenchyma. Unfortunately, NACT was ceased prematurely after three cycles due to toxicity. 

**Figure 4:**
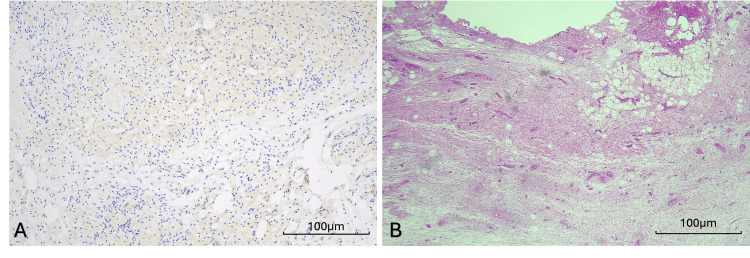
(A) Cytokeratin AE1/AE3 staining and (B) H&E staining Histopathological slides of interest demonstrate the tumour bed from the left axilla, with evidence of treatment-related changes, but no residual tumour. Scale bar represents 100 µm; magnification 20×, for figures (A) and (B). H&E, haematoxylin and eosin

A decision was made for a bilateral mastectomy with bilateral ALND. Histopathological results revealed no residual malignancy in the bilateral breasts, no residual carcinoma in 27 lymph nodes in the right axilla (one node with treatment-related changes), and no residual carcinoma in nine lymph nodes in the left axilla (two nodes with treatment-related changes) (Figure 4).

The patient was subsequently referred for adjuvant radiation to the left supraclavicular and clavipectoral nodes. However, prior to commencement of radiation therapy, the patient developed a progressive three-week history of worsening balance, headaches, and nausea. A CT brain demonstrated a new 38 mm left cerebellar lesion, consistent with leptomeningeal disease. This was deemed unresectable, and, after further discussions with the Community Palliative Care Team, the patient and her family subsequently decided to transition to comfort care, and she subsequently passed away.

## Discussion

Breast cancer is the most common cause of malignancy and the second leading cause of cancer-related death in women globally [5,6]. Screening for breast cancer includes the early detection of breast cancer, with mammography as the gold-standard imaging modality [6]. In Australia, screening mammography is recommended for all women aged 50 to 74, biennially [7]. While most breast lesions can be identified clinically or via imaging, very rarely, an OBC may occur.

OBC is a rare presentation of breast cancer, characterised by breast carcinoma that has metastasised to the axillary lymph nodes, with the absence of radiological disease in the breast [1,2]. Most commonly, patients with OBC present with new axillary lymphadenopathy as their initial presentation [2]. Apart from the conventional imaging modalities of an ultrasound and a mammogram, a breast MRI is often recommended to improve the detection of occult primary breast cancer [1]. In addition, breast MRI is also useful to assess the extent of disease, evaluate response to neoadjuvant treatment, and assess lymph nodes and occult primary tumours [8].

Historically, the surgical management of the breast in OBC was a mastectomy, while ALND remains the mainstay of surgical management of the axilla [3]. The current guidelines by the National Comprehensive Cancer Network (NCCN) for OBC recommend mastectomy with ALND, or ALND and whole-breast radiotherapy; however, treatment of the ipsilateral breast remains contentious due to the various prognoses in clinical practice [2]. Interestingly, a recent meta-analysis conducted by Wang et al. in 2023, involving 3,476 patients across 13 studies, demonstrated the role of NACT in the treatment of OBC, improving patient survival [2]. In addition, the evolving role of NACT may also allow for de-escalation of axillary surgery - though further long-term research is required to support this hypothesis [3].

CAM in breast cancer is another rare occurrence, with differentials including a contralateral metastasis from the primary breast cancer, metastasis from a separate primary ipsilateral breast cancer, or metastasis from another primary cancer of extramammary origin [4]. Given the presence of metastatic breast cancer to the contralateral axilla, CAM is regarded as a stage IV disease, with the classification of M1 [9].

However, the occurrence of CAM could be due to differences in the usual lymphatic drainage pathway - thus suggesting that CAM could be classified as a variable presentation of locoregional, rather than systemic, disease [4,10]. Treatment options for CAM favour chemoradiotherapy, while contralateral mastectomy is not routinely recommended, except in specific scenarios, such as CAM with different pathological features from the primary malignancy [4].

## Conclusions

In conclusion, OBC and CAM are both rare and complex presentations of breast cancer. This case highlights the complexities and diagnostic dilemmas of OBC and CAM. It is important for clinicians to have a high index of suspicion when there is evidence of metastatic breast cancer, regardless of a clear primary breast malignancy. While an ALND remains the mainstay of surgical management of the axilla for OBC, there are yet to be consensus guidelines on the management of the breast. CAM is a rare presentation in breast cancer, with controversy surrounding its classification as a locoregional rather than systemic disease.
